# Is tracheostomy a better choice than translaryngeal intubation for critically ill patients requiring mechanical ventilation for more than 14 days? A comparison of short-term outcomes

**DOI:** 10.1186/s12871-015-0159-9

**Published:** 2015-12-15

**Authors:** Wei-Chieh Lin, Chang-Wen Chen, Jung-Der Wang, Liang-Miin Tsai

**Affiliations:** Department of Internal Medicine, National Cheng Kung University Hospital, College of Medicine, National Cheng Kung University, No. 138 Sheng-Li Road, Tainan, 704 Taiwan; Department of Public Health, College of Medicine, National Cheng Kung University, No. 1 University Road, Tainan, 701 Taiwan

**Keywords:** Intensive care unit, Mortality, Tracheostomy, Weaning, Prolonged mechanical ventilation

## Abstract

**Background:**

Tracheostomy is recommended for patients receiving mechanical ventilation (MV) for 14 days or more in the intensive care unit (ICU). Nevertheless, many patients undergoing prolonged MV remain intubated via the translaryngeal route. The aim of this study was to examine the influence of tracheostomy and persistent translaryngeal intubation on short-term outcomes in patients mechanically ventilated for ≥14 days.

**Methods:**

A retrospective study was conducted using the admissions database of a 75-bed ICU from January 1, 2012, to December 31, 2012. Patients who required prolonged MV without tracheostomy at the time of initiation of a ventilator were included. The outcomes were successful weaning, and ICU and in-hospital death. Cox models were constructed to calculate the influence of tracheostomy on the outcome measures while adjusting for other potentially confounding factors.

**Results:**

Of the 508 patients requiring prolonged MV, 164 were tracheostomized after a median 18 days of MV. Patients in whom translaryngeal intubation was maintained had significantly higher ICU (42.7 % versus 17.1 %, *p* <0.001) and in-hospital (54.1 % versus 22.0 %, *p* <0.001) mortality rates, and a significantly lower successful weaning rate (40.4 % versus 68.9 %, *p* <0.001). The results were consistent after matching for the propensity score of performing tracheostomy. Furthermore, a time-dependent covariate Cox model showed that a tracheostomy was independently associated with lower in-hospital mortality (adjusted hazard ratio [aHR], 0.26; 95 % confidence interval [CI], 0.18–0.39) and higher successful weaning rate (aHR, 2.05; 95 % CI, 1.56–2.68).

**Conclusions:**

Tracheostomy is associated with lower in-hospital mortality and higher successful weaning rates in ICU patients receiving prolonged MV. However, the cost-effectiveness and long-term outcomes of tracheostomy for this cohort require further study.

**Electronic supplementary material:**

The online version of this article (doi:10.1186/s12871-015-0159-9) contains supplementary material, which is available to authorized users.

## Background

The number of patients requiring prolonged mechanical ventilation (PMV) in the intensive care unit (ICU) is increasing [1]. These patients consume a substantial amount of healthcare resources [2] and most have poor short- and long-term outcomes [2, 3]. Tracheostomy is thought to provide several advantages over translaryngeal intubation in patients undergoing PMV, such as the promotion of oral hygiene and pulmonary toilet, improved patient comfort, decreased airway resistance, accelerated weaning from mechanical ventilation (MV) [4], the ability to transfer ventilator-dependent patients from the ICU to step-down facilities [5] and a reduced risk of developing ventilator-associated pneumonia (VAP) [6]. Although tracheostomy is customarily performed to facilitate care of patients requiring PMV, physician practices regarding tracheostomy differ widely [7]; furthermore, a significant proportion of patients or family members decline to give consent for tracheostomy, even when long-term ventilator dependence is expected. The reported rates of tracheostomy range from 5–24 % and time to tracheostomy ranges from 9–12 days [8]. Three meta-analyses of studies examining the role of tracheostomy in critically ill patients receiving MV have failed to demonstrate any benefits of “early” tracheostomy on survival, length of ICU or hospital stay, or duration of MV, compared with those undergoing “late” tracheostomy or prolonged translaryngeal intubation [9–11]. It is important to note, however, that two of these studies used ≤7 days after translaryngeal intubation as the definition of “early” tracheostomy [10, 11], while the other used ≤10 days [9].

Consequently, most experts recommend that tracheostomy be deferred for at least 10–14 days after translaryngeal intubation to ensure that ongoing MV is indeed required [4, 11, 12]. Currently, most clinicians view 1–2 weeks after intubation as the most appropriate timing for tracheostomy [9]. Nonetheless, many patients still undergo MV via a translaryngeal endotracheal tube for more than 2 weeks. We undertook a study to examine the outcomes of patients undergoing MV for at least 14 days in the ICU via persistent translaryngeal intubation or tracheostomy. We hypothesized that tracheostomy would be associated with an increased rate of successful weaning and reduced ICU and in-hospital mortality in critically ill patients requiring PMV compared with persistent translaryngeal intubation. The aim of this study was to evaluate the effect of tracheostomy on the outcome of patients receiving PMV (≥14 days) in our medical-surgical ICU.

## Methods

Conduct of the study was approved by the ethics committee of National Cheng Kung University Hospital (reference A-ER-103-284-T). It was judged that the retrospective observational study design posed no risks to patients. The need for informed consent was waived, as all data were anonymized and patient identification numbers were encrypted.

### Study design

This was a retrospective cohort study using anonymized data from an ICU clinical information system (IntelliVue Clinical Information Portfolio, Philips) derived from a single center. The database included vital signs and ventilation status recorded automatically from monitors and ventilators, laboratory data retrieved automatically from our hospital’s central laboratory report system, fluid balance, and clinical and nursing procedures. Demographic data recorded routinely at ICU admission by supervising nurses included age, sex and the Acute Physiology and Chronic Health Evaluation II (APACHE II) score. Two analytical models were constructed: a multivariable time-dependent Cox model, and propensity score matching for performing tracheostomy that adjusted for potential confounding factors to evaluate the effect of tracheostomy on the outcomes of patients with PMV.

### Patients and setting

Data were obtained from patients admitted to the 75-bed adult ICU at the tertiary referral center of southern Taiwan between January 1, 2012, and December 31, 2012. Patients who were older than 16 years and received MV ≥24 h were screened, those who underwent MV for at least 14 days were included in the study. Patients with a tracheostomy present at the time of initiation of MV were excluded. For patients who had more than one ICU admission with MV ≥14 days during the study period, only the first admission was included in the analyses to avoid the potential selection bias of including patients without tracheostomy in whom the risk of death was higher.

In our clinical practice, tracheostomy is normally undertaken after an episode of failed extubation or reintubation, in the presence of unrelieved upper airway obstruction, when airway protection or regular pulmonary toilet is indicated, when PMV is needed, or for the avoidance of the complications of prolonged translaryngeal intubation. All tracheostomies are formed surgically. The decision to perform a tracheostomy is at the discretion of the attending physician, but only after informed consent has been obtained from the patient, their next of kin or their legal representative.

### Variables

Study variables included in the analyses were: age; sex; comorbidities; Charlson comorbidity index (CCI) score; APACHE II score within 24 h of ICU admission; ICU type (medical or surgical ICU); presence of a do-not-resuscitate (DNR) order; time to tracheostomy (if performed); diagnosis at ICU admission; requirement for non-invasive ventilation (NIV) after extubation; duration of MV and successful weaning from MV; ICU and hospital lengths of stay; and ICU and in-hospital mortality. Laboratory data including blood urea nitrogen (BUN), serum creatinine concentration, blood gas analysis, and white blood cell (WBC) and platelet counts were recorded. In this observational study, we could not mandate that routine laboratory tests be performed at particular times. We therefore recorded variables obtained on days 1 to 5 of ICU admission (week 1, w1) and days 8 to 12 (week 2, w2) to ensure that all data were available for the analyses. The least favorable value of each variable was chosen for analysis if more than two values existed within the same time period. ICU admission diagnosis and comorbidities were identified according to the International Classification of Diseases, ninth revision and categorized as shown in the supplementary material (Additional file 1). Non-invasive ventilation was initiated after extubation as needed, if it was judged that patients were at high risk of extubation failure [13–16]. Successful weaning was recorded when MV was not required again after discontinuation during hospitalization, regardless of outcome.

### Statistical analysis

We categorized the patients into a tracheostomy group and a translaryngeal tube group. Continuous variables are expressed as the mean ± standard deviation (SD), or the median and interquartile range (IQR). Categorical variables are expressed as frequencies and proportions (%). Continuous variables were compared with Student’s *t* test or the Mann–Whitney *U* test (for skewed data); categorical variables were compared with the chi-squared test or Fisher’s exact test, as necessary. Levels of significance are expressed as *p* values; *p* <0.05 was considered statistically significant. Demographic, clinical and laboratory values were used to determine whether tracheostomy was independently associated with both in-hospital mortality and successful weaning. As the formation of a tracheostomy was considered to be a time-dependent covariate, a multivariable time-dependent Cox model was constructed. Median values were used to categorize continuous variables for Cox models. The factors with a *p* value <0.1 in the univariate analysis, together with age and sex regardless of the univariate *p* value, were entered in the Cox models. The results are presented as adjusted hazard ratios (aHR) with 95 % confidence intervals (CIs). The impact of tracheostomy on outcomes was assessed using a propensity score for a case-matched comparison to deal with significant heterogeneity between patients undergoing tracheostomy or persistent translaryngeal intubation in a retrospective cohort study (Table 1). The case-matched comparison was performed as previously described [17]. All univariable predictors of tracheostomy with *p* <0.25 together with age and sex were entered into the multivariable model predicting tracheostomy. Stepwise logistic regression was performed to remove covariates that had multivariable *p* values of >0.25. A propensity score for undergoing tracheostomy was calculated for each patient using the coefficients of the final regression equation. Then, each tracheostomy patient was matched with a single translaryngeal intubated patient who had a similar propensity score (within 0.1 on a scale from 0 to 1). When more than one matched patient was identified for a case, the closest admission date was used to select the matched patient. We also performed a sensitivity analysis using a more stringent propensity score matching within 0.05. The matched Wilcoxon test, the McNemar test, and mixed model or general estimation equations were used for case-match study comparisons. Analyses were performed using SPSS, version 20 for Windows (SPSS Inc., Chicago, IL, USA).

**Table 1:** Demographic and clinical characteristics of patients mechanically ventilated for at least 14 days

	Translaryngeal tube (*n* = 344)	Tracheostomy (*n* = 164)	*p* value
Age, years	67 ± 14	62 ± 18	0.416
Male, *n* (%)	203 (59)	110 (67)	0.099
MICU, *n* (%)	237 (69)	70 (43)	<0.001
APACHE II score	23.1 ± 7.8	23.2 ± 7.5	0.446
Do-not-resuscitate order, *n* (%)	157 (46)	39 (24)	<0.001
NIV after extubation, *n* (%)	79 (23)	24 (15)	0.039
Diagnosis of ICU admission, *n* (%)			
Pneumonia	190 (55)	94 (57)	0.729
Sepsis	164 (48)	68 (42)	0.223
Cardiovascular or vascular disorder	39 (11)	12 (7)	0.211
Trauma	20 (6)	23 (14)	0.003
Brain disorder	99 (29)	76 (46)	<0.001
Burn	5 (2)	0 (0)	0.181
Gastrointestinal Disorder	103 (30)	51 (31)	0.872
CCI score	2 (1–3)	2 (0–3)	0.017
Comorbidities, *n* (%)			
Diabetes	121 (35)	66 (40)	0.313
Chronic lung disease	75 (22)	37 (23)	0.938
Chronic heart disease	91 (27)	29 (18)	0.039
Chronic liver disease	49 (14)	12 (7)	0.036
Chronic renal disease	91 (27)	30 (18)	0.056
Malignancy	107 (31)	44 (27)	0.378
Connective tissue disease	14 (4)	1 (1)	0.045
Neuromuscular disease	19 (6)	10 (6)	0.955
PaO_2_/FiO_2_			
w1	290.9 ± 136.6	266.6 ± 132.0	0.245
w2	307.9 ± 101.2	293.6 ± 110.0	0.151
WBC (× 10^3^/μl)			
w1	12.3 (9.6–16.6)	10.8 (8.4–15.9)	0.808
w2	10.5 (7.9–12.8)	11.5 (7.9–14.9)	0.638
Platelet (× 10^3^/μl)			
w1	193.0 (129.0–233.0)	148.0 (102.5–195.3)	0.076
w2	181.0 (107.0–240.0)	143.0 (88.0–263.5)	0.066
BUN (mg/dl)			
w1	18.0 (13.0–40.0)	28.5 (14.3–49.8)	0.146
w2	35.0 (24.0–65.0)	44.0 (22.0–65.3)	0.025
Creatinine (mg/dl)			
w1	1.1 (0.8–2.0)	1.0 (0.8–2.0)	0.014
w2	1.0 (0.6–2.0)	1.0 (0.6–2.4)	0.020

Data are presented as mean ± standard deviation or median (interquartile range) unless otherwise stated

*Abbreviations*: *APACHE II* Acute physiology and chronic health evaluation II, *CCI* Charlson comorbidity index, *MICU* Medical intensive care unit, *NIV* Non-invasive ventilation, *BUN* Blood urea nitrogen, *PaO*_*2*_ */FiO*_*2*_ ratio of the partial pressure of arterial oxygen to the fraction of inspired oxygen, *WBC* White blood cell count, *w1*, Data collected within days 1–5 after ICU admission, *w2* Data collected within days 8–12 after ICU admission

## Results

**Fig. 1:**
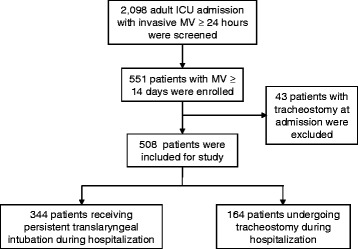
Flow diagram of the study population. *Abbreviations*: *ICU* Iintensive care unit, *MV* Mechanical ventilation

MV was required for at least 24 h in 2,098 adult ICU admissions during the study period. Of these, 551 patients (26.3 %) who received MV for at least 14 days were screened, and those who already had a tracheostomy tube *in situ *at the initiation of MV were excluded. The remaining 508 patients were included in the analyses. During their ICU admission, 164 patients (32.3 %) underwent a tracheostomy. The remaining 344 patients (67.7 %) did not undergo a tracheostomy during hospitalization (Fig. 1). For those who received tracheostomy, the median time between endotracheal intubation and insertion of a tracheostomy tube was 18 days (IQR, 13–25 days). The overall ICU and in-hospital mortality rates were 34.4 % and 43.7 %, respectively.

The baseline characteristics of the study population are summarized in Table 1. A greater proportion of translaryngeal intubated patients were admitted to the medical ICU, had a current DNR order, had a higher CCI score, had chronic heart, liver or connective tissue disease, and required NIV after extubation, but a smaller proportion had brain disorders or had sustained trauma. There were no significant differences in the ratio of the partial pressure of arterial oxygen to the fraction of inspired oxygen (PaO_2_/FiO_2_), WBC count or platelet count between the groups. The serum concentrations of BUN (w2) and creatinine (w1 and w2) were significantly lower in the tracheostomy group than the translaryngeal tube group.

Table 2 shows a comparison of the short-term outcomes between the groups. Compared with those with a tracheostomy, translaryngeal intubated patients had significantly higher ICU (42.7 % versus 17.1 %, *p* <0.001) and in-hospital (54.1 % versus 22.0 %, *p* <0.001) mortality rates, lower weaning rate (40.4 % versus 68.9 %, *p* <0.001) and significantly shorter median duration of MV (26 days versus 37 days, *p* <0.001), ICU length of stay (25 days versus 40 days, *p* <0.001) and hospital length of stay (30 days versus 59 days, *p* <0.001). In addition, translaryngeal intubated patients were transferred earlier to lower level regional hospitals compared with tracheostomized patients (28 days versus 54 days, *p* <0.001).

**Table 2:** Clinical outcomes of patients receiving mechanical ventilation for at least 14 days

	Translaryngeal tube (*n* = 344)	Tracheostomy (*n* = 164)	*p* value
Duration of MV, days	26 (21–35)	37 (25–51)	<0.001
Weaning rate, n (%)	139 (40 %)	113 (69 %)	<0.001
Transition to regional hospitals, n (%)	62 (18 %)	40 (24 %)	0.120
Time to transition, days	28 (21–40)	54 (44–80)	<0.001
ICU length of stay, days	25 (20–33)	40 (25–55)	<0.001
Hospital length of stay, days	30 (22–45)	59 (45–94)	<0.001
ICU mortality, n (%)	147 (43 %)	28 (17 %)	<0.001
Hospital mortality, n (%)	186 (54 %)	36 (22 %)	<0.001

Data are presented as median (interquartile range) unless otherwise stated

*Abbreviations*: *MV* Mechanical ventilation, *ICU* Intensive care unit

Multivariable time-dependent Cox regression modeling showed that performing a tracheostomy was associated with a significantly lower risk of in-hospital death (aHR 0.26, 95 % CI 0.18–0.39, *p* <0.001) and a significantly higher chance of successful weaning (aHR 2.05, 95 % CI 1.56–2.68, *p* <0.001; Table 3). The other independent factors significantly associated with in-hospital mortality included a DNR order, undergoing NIV after extubation, malignancy and a PaO_2_/FiO_2_ ratio >282 (w2) (Table 3). The other factors independently associated with successful weaning included a DNR order, sepsis, chronic lung disease, APACHE II score >23, a PaO_2_/FiO_2_ ratio >282 (w2) and a platelet count >140 × 10^3^/μl (w2) (Table 3).

**Table 3:** Factors associated with in-hospital mortality and successful weaning in patients receiving mechanical ventilation for at least 14 days

	Hospital mortality		Successful weaning	
	aHR (95 % CI)	*p* value	aHR (95 % CI)	*p* value
Performing tracheostomy	0.26 (0.18–0.39)	<0.001	2.05 (1.56–2.68)	<0.001
Do-not-resuscitate order	2.55 (1.92–3.38)	<0.001	0.53 (0.39–0.72)	<0.001
NIV after extubation	0.50 (0.35–0.71)	<0.001		
Malignancy	1.33 (1.01–1.75)	0.044		
Sepsis			0.61 (0.47–0.80)	<0.001
Chronic lung disease			0.68 (0.50–0.93)	0.016
APACHE II score > 23			0.71 (0.55–0.93)	0.012
PaO_2_/FiO_2_ > 282 (w2)	0.73 (0.56–0.96)	0.023	1.34 (1.04–1.73)	0.023
Platelet > 140 × 10^3^/μl (w2)			1.37 (1.05–1.79)	0.020

*Abbreviations*: *aHR* Adjusted hazard ratio, *APACHE II* Acute physiology and chronic health evaluation II, *CI* Confidence interval, *NIV* Noninvasive ventilation, *OR* Odds ratio, *PaO*_*2*_ */FiO*_*2*_ Ratio of the partial pressure of arterial oxygen to the fraction of inspired oxygen, *w2* Data collected within days 8–12 after ICU admission

In view of the significant baseline heterogeneity between the tracheostomy and translaryngeal intubation groups (see Table 1), we performed a case-matched comparison using the propensity score-based algorithm. As shown in Table 4, except for pneumonia, no significant differences were found between the two groups concerning ICU admission or clinical characteristics in week 1 or week 2. Similar to the findings before matching, tracheostomized patients had significantly lower ICU and hospital mortality rates, a higher successful weaning rate, longer duration of MV, ICU and hospital stays, and later transition to regional hospitals (Table 4). In a sensitivity analysis to match the propensity score within 0.05, 139 cases for each group were matched, and the results were consistent with those described above.

**Table 4:** Case-matched study: demographic and clinical characteristics of patients mechanically ventilated for at least 14 days

	Translaryngeal tube (*n* = 164)	Tracheostomy (*n* = 164)	*p* value
Age, years	68 (54–77)	64 (51–74)	0.749
Male, *n* (%)	108 (66)	110 (67)	0.899
MICU, *n* (%)	87 (53)	94 (57)	0.464
APACHE II score	24.3 ± 7.4	23.2 ± 7.5	0.448
Do-not-resuscitate order	44 (27)	39 (24)	0.568
NIV after extubation, *n* (%)	26 (16)	24 (15)	0.871
Diagnosis of ICU admission, *n* (%)			
Pneumonia	75 (46)	94 (57)	0.033
Sepsis	68 (42)	68 (42)	1.000
Cardiovascular or vascular disorder	13 (8)	12 (7)	1.000
Trauma	17 (10)	23 (14)	0.361
Brain disorder	70 (43)	76 (46)	0.571
Gastrointestinal disorder	42 (26)	51 (31)	0.328
CCI score	2 (1–3)	2 (0–3)	0.836
Comorbidities, *n* (%)			
Diabetes	55 (34)	66 (40)	0.235
Chronic lung disease	36 (22)	37 (23)	1.000
Chronic heart disease	30 (18)	29 (18)	1.000
Chronic liver disease	14 (9)	12 (7)	0.839
Chronic renal disease	35 (21)	30 (18)	0.575
Malignancy	44 (27)	44 (27)	1.000
Connective tissue disease	1 (1)	1 (1)	1.000
Neuromuscular disease	8 (5)	10 (6)	0.804
PaO_2_/FiO_2_			
w1	302.1 ± 137.7	266.6 ± 132.0	0.899
w2	312.0 ± 101.9	293.6 ± 110.0	0.734
WBC (× 10^3^/μl)			
w1	11.9 (9.0–14.4)	10.8 (8.4–15.9)	0.901
w2	10.7 (8.1–12.8)	11.5 (7.9–14.9)	0.449
Platelet (× 10^3^/μl)			
w1	181.0 (122.0–226.0)	148.0 (102.5–195.3)	0.516
w2	183.3 ± 100.6	170.1 ± 99.2	0.978
BUN (mg/dl)			
w1	18.0 (13.0–35.0)	28.5 (14.3–49.8)	0.774
w2	38.0 (24.0–69.0)	44.0 (22.0–65.3)	0.706
Creatinine (mg/dl)			
w1	1.1 (0.8–2.5)	1.0 (0.8–2.0)	0.111
w2	1.3 (0.7–2.4)	1.0 (0.6–2.4)	0.327
Transition to regional hospitals, n (%)^a^	39 (24)	40 (24)	0.808
Time to transition, days^b^	27 (21–45)	54 (44–80)	0.009
Duration of MV, days^b^	28 (21–35)	37 (25–51)	<0.001
Weaning rate, *n* (%)^a^	65 (40)	113 (69)	<0.001
ICU length of stay, days^b^	27 (21–34)	40 (25–55)	<0.001
Hospital length of stay, days^b^	30 (23–46)	59 (45–94)	<0.001
ICU mortality, n (%)^a^	63 (38)	28 (17)	<0.001
Hospital mortality, n (%)^a^	76 (46)	36 (22)	<0.001

Data are presented as median (interquartile range) or mean ± standard deviation unless otherwise stated

^a^Compared with general estimation equation analysis, adjusted for pneumonia

^b^Compared with mixed-model analysis, adjusted for pneumonia

*Abbreviations*: *APACHE II* Acute physiology and chronic health evaluation II, *BUN* Blood urea nitrogen, *CCI* Charlson comorbidity index, *MICU* Medical intensive care unit, *MV* Mechanical ventilation, *NIV* Noninvasive ventilation, *ICU* Intensive care unit, *PaO*_*2*_ */FiO*_*2*_ Ratio of the partial pressure of arterial oxygen to the fraction of inspired oxygen, *WBC* White blood cell count, *w1* Data collected within days 1–5 after ICU admission, *w2* Data collected within days 8–12 after ICU admission

## Discussion

We found that patients requiring PMV (≥14 days) who did not undergo tracheostomy had significantly higher ICU and in-hospital mortality, and were less likely to be successfully weaned, after carefully controlling for potential demographic and clinical confounders. Interestingly, translaryngeal intubated patients had significantly shorter durations of MV and ICU and hospital stays than patients who underwent a tracheostomy. To the best of our knowledge, this is the first report to have compared the short-term outcomes of patients who received MV for at least 14 days and either underwent tracheostomy or remained intubated by the translaryngeal route for a prolonged period.

We also found that the ICU type, a DNR order, undergoing NIV after extubation, brain disorders and trauma as the cause of ICU admission, renal function, CCI score and comorbidities such as chronic heart, liver and connective tissue diseases were associated with the rate and timing of tracheostomy, findings that are consistent with a previous report [17]. Our finding that tracheostomy was independently associated with reduced ICU and in-hospital mortality rates is also consistent with some previous reports [17, 18], but not others [19, 20]. Clec'h et al. [19] reported that tracheostomy had no positive influence on survival when performed in unselected mechanically ventilated patients. Their cohort included patients requiring MV for at least 2 days but nearly half received MV for <15 days, and therefore their findings may have been biased by the presence of a subgroup more likely to have been weaned from MV without the need for tracheostomy [9]. In contrast, our study included only those patients receiving MV for ≥14 days, a cohort in which failed extubation and severe comorbidities were more common; a greater proportion would be expected to require long-term ventilatory support and would be more likely to benefit from tracheostomy [4]. Similarly, Trouillet and colleagues [20] compared patients undergoing tracheostomy after 5 days of ventilatory support with those intubated by the translaryngeal route for a prolonged period, and found that tracheostomy did not affect mortality; however, they included patients ventilated after cardiac surgery who had failed weaning trials at day 4 of MV and 27 % of the patients in the prolonged translaryngeal intubation group eventually underwent a tracheostomy.

In our study, the substantially reduced risk of death (aHR 0.26) in the tracheostomy group could in part be explained by the fact that tracheostomy is associated with a decreased risk for VAP in patients requiring PMV [6]. In tracheostomized patients, tracheostomy allows the vocal cords to close, reduces aspiration of oropharyngeal secretions, reduces bacterial biofilm formation along the inside of the tracheotomy cannula and facilitates weaning from MV. All these factors probably result in a reduced risk for VAP [6]. Another potential explanation is that ICU physicians may be adept at selecting candidates for tracheostomy based on the highest probability of survival, and therefore may provide more aggressive treatment for these patients while being more likely to offer conservative or palliative treatment to those intubated by the translaryngeal route for a prolonged period. In a retrospective study, it is also possible that we might have missed some important confounding factors associated with the decision to undertake tracheostomy that might also affect outcomes, even when sophisticated adjustment methods such as multivariable analyses or propensity score-based nested case–control studies are used.

Aside from tracheostomy, we identified a DNR order, NIV after extubation, malignancy and a PaO_2_/FiO_2_ ratio (w2) as the factors independently associated with in-hospital mortality in patients receiving MV for ≥14 days. There is a body of evidence that using NIV after extubation improves survival rate in high-risk patients [13–16]; NIV is commonly employed in our clinical practice to prevent post-extubation respiratory failure. Malignancy is recognized as a predictor of poor outcome in patients requiring PMV [3]. Increased PaO_2_/FiO_2_ ratio in week 2 might simply reflect a favorable response to treatment, leading to better outcomes. Furthermore, we found that tracheostomy was associated with increased successful weaning rates. In contrast, Wu et al. [18] reported that tracheostomy has no effect on successful weaning. This discrepancy might be explained by a difference in study setting (ours was conducted in an ICU while theirs took place in a specialist respiratory care center). Moreover, tracheostomy has been reported to contribute to facilitation of weaning from MV by decreasing airflow resistance and the associated work of breathing, allowing clinicians to be more aggressive in their weaning strategies and reducing their concerns about sedation and reintubation [4]. In addition, we found that a DNR order, sepsis, chronic lung disease, APACHE II score, PaO_2_/FiO_2_ ratio (w2) and platelet count (w2) were independently associated with weaning success. These findings are similar to that of previous studies regarding the prediction of weaning or extubation failure [16]. Platelet count has also been reported to be associated with successful weaning in patients requiring PMV after cardiac surgery [21]. The relationship between existence of a current DNR order and poor outcomes can be explained by the presence of irreversible and terminal diseases.

There have been reports that early tracheostomy was not associated with a reduced length of ICU stay, hospital stay or duration of MV [9, 10]. Indeed, consistent with previous studies [17–19], we found that tracheostomy increased the duration of MV and length of ICU and hospital stays compared with translaryngeal intubation. These observations may be partially explained by the relatively long median time before tracheostomy (18 days) in the tracheostomy group. The shorter duration of MV, and shorter length of ICU and hospital stays in the translaryngeal intubation group probably reflect these patients’ higher mortality rate and earlier death (30 days compared with 61 days) or transition to lower-level regional hospitals.

Our study had some limitations. First, we did not record data regarding the effects of inadvertent extubation on the outcomes of the translaryngeal intubated group, tracheostomy complications, the incidence of extubation failure or the rate of VAP. All these factors may be associated with patient morbidity, mortality and successful weaning. We did not record serial ICU scores (such as the daily Sequential Organ Failure Assessment), which may have more accurately reflected the severity of illness at the time of tracheostomy (after a median of 18 days of MV) than the APACHE II score in the first 24 h of ICU admission [17]. Second, our study was undertaken retrospectively; this may have caused us to miss important confounders relevant to the results, while accounting for the significant heterogeneity between the translaryngeal intubation group and the tracheostomy group. Consequently, we cannot completely rule out the possibility that clinical practice on the ICU tends to select patients with the highest likelihood of survival for tracheostomy, although we matched for propensity scores, adjusted for potential confounders and performed a sensitivity analysis in which even more stringent propensity score matching was employed that yielded the same results. Third, we did not assess the long-term outcomes after hospital discharge. Further study is needed to determine whether there are long-term benefits of tracheostomy on outcomes compared with translaryngeal intubation. A prospective, randomized controlled study will be needed to eliminate the biases described above.

## Conclusions

Tracheostomy was independently associated with reduced ICU and in-hospital mortality, and increased successful weaning rate, for critically ill patients requiring MV for at least 14 days. Tracheostomized patients had a longer duration of MV and length of ICU and hospital stays compared with patients who remained intubated by the translaryngeal route for a prolonged period. The cost-effectiveness and long-term outcomes of tracheostomy for critically ill patients requiring PMV require further study.

### Key messages

- For patients requiring MV for at least 14 days, tracheostomy was significantly associated with reduced ICU and hospital mortality and increased successful weaning rate. The results were consistent after matching for the propensity score of performing tracheostomy.
- A time-dependent covariate Cox model showed that tracheostomy was independently predictive of lower in-hospital mortality and higher successful weaning rate.
- Our finding that tracheostomized patients required longer periods of MV, and longer ICU stay and hospitalization warrants further study to evaluate the cost-effectiveness and long-term outcomes of tracheostomy for critically ill patients requiring PMV.

## Additional file

Additional file 1: Table S1. Definition of primary ICU admission diagnosis and comorbidities in our cohort. (PDF 60 kb)

## Abbreviations

APACHE: Acute physiology and chronic health evaluation
BUN: blood urea nitrogen
CCI: Charlson comorbidity index
CI: confidence interval
HR: hazard ratio
ICU: intensive care unit
IQR: interquartile range
MV: mechanical ventilation
NIV: noninvasive ventilation
OR: odds ratio
PaO_2_/FiO_2_: ratio of the partial pressure of arterial oxygen to the fraction of inspired oxygen
PMV: prolonged mechanical ventilation
WBC: white blood cell
