# Supplementary material for: Is tracheostomy a better choice than translaryngeal intubation for critically ill patients requiring mechanical ventilation for more than 14 days? A comparison of short-term outcomes
Source: BMC Anesthesiol. 2015 Dec 15;15:181. doi: 10.1186/s12871-015-0159-9 (PMC4681017; doi:10.1186/s12871-015-0159-9)
Supplement: Additional file 1: Table S1. — Definition of primary ICU admission diagnosis and comorbidities in our cohort. (PDF 60 kb) [file 12871_2015_159_MOESM1_ESM.pdf]

Additional file 1

Table 1: Definition of primary ICU admission diagnosis and comorbidities in our cohort

Category	Definition
Diagnosis of ICU admission	
Pneumonia	All pulmonary infections, and tuberculosis.
Sepsis	All septic conditions at ICU admission.
Cardiovascular or vascular disorders	Acute coronary syndromes, acute decompensated heart failure; cardiogenic shock, arrhythmias, and aortic dissection.
Trauma	—
Brain disorder	Status epilepticus, intracranial hemorrhage, stroke, anoxic brain damage, encephalitis, meningitis, intracranial abscess, and tumor.
Burns	—
Gastrointestinal disorders	Gastrointestinal bleeding, perforation, obstruction, and adhesions.
Comorbidities	
Diabetes	—
Chronic lung disease	Obstructive and restrictive lung diseases, old pulmonary tuberculosis, and bronchiectasis.
Chronic heart disease	Congestive heart failure, valvular heart disease, and coronary artery disease.
Chronic liver disease	Hepatic cirrhosis and chronic hepatitis.
Chronic renal disease	End-stage renal disease, nephrotic and nephritic syndrome, and other chronic renal insufficiencies.
Malignancy	Hematological malignancy and solid tumor
Connective tissue disease	Systemic lupus erythematosus, rheumatoid arthritis, dermatomyositis, and other connective tissue diseases.
Neuromuscular disease	Myasthenia gravis, Parkinson's disease, amyotrophic lateral sclerosis, and other neuropathy or myopathy.
